# Targeting the Tumor Microenvironment: A Literature Review of the Novel Anti-Tumor Mechanism of Statins

**DOI:** 10.3389/fonc.2021.761107

**Published:** 2021-11-11

**Authors:** Peng-Fei Zhu, Ming-Xing Wang, Zhe-Ling Chen, Liu Yang

**Affiliations:** ^1^ Cancer Center, Department of Medical Oncology, Zhejiang Provincial People’s Hospital, People’s Hospital of Hangzhou Medical College, Hangzhou, China; ^2^ Graduate School of Clinical Medicine, Bengbu Medical College, Bengbu, China

**Keywords:** statin, HMG-CoA, tumor microenvironment, pyroptosis, ferroptosis, autophagy

## Abstract

Statins is widely used in clinical practice as lipid-lowering drugs and has been proven to be effective in the treatment of cardiovascular, endocrine, metabolic syndrome and other diseases. The latest preclinical evidence shows that statins have anti-proliferation, pro-apoptotic, anti-invasion and radiotherapy sensitization effects on tumor cells, suggesting that statins may become a new type of anti-tumor drugs. For a long time, mevalonate pathway has been proved to play a supporting role in the development of tumor cells. As an effective inhibitor of mevalonate pathway, statins have been proved to have a direct auxiliary anti-tumor effect in a large number of studies. In addition, anti-tumor effects of statins through ferroptosis, pyroptosis, autophagy and tumor microenvironment (TME) have also been gradually discovered. However, the specific mechanism of the antitumor effect of statins in the tumor microenvironment has not been clearly elucidated. Herein, we reviewed the antitumor effects of statins in tumor microenvironment, focusing on hypoxia microenvironment, immune microenvironment, metabolic microenvironment, acid microenvironment and mechanical microenvironment.

## Introduction

Statins are potent inhibitors of 3-hydroxy-3-methylglutaryl coenzyme A (HMG-CoA) reductase, which is the rate-controlling enzyme of the mevalonate pathway (1). Statins have a pleiotropic effect on cell survival, cell adhesion, migration, proliferation, immune regulation, antioxidant activity, endothelial function and angiogenesis (2). Dysregulation of the mevalonate (MVA) pathway has been found in a variety of tumors, including pancreatic, breast, liver and statins as inhibitors of MVA pathway might be useful for cancer prevention and treatment and statins reduces cancer-related mortality among cancer patients (3, 4). Several mechanisms may be involved in dysregulation of this pathway. They include P53 mutation, a mutation in HMG-CoA reductase and sterol-regulatory element binding proteins (SREBPs) cleavage-activating protein (SCAP) as its regulator, PKB/Akt activation, decreased adenosine monophosphate–activated protein kinase (AMPK) activation, and activation of transcription factors such as: SREBP and Hypoxia-inducible factor-1 (HIF-1) (4). Recent years, with the continuous innovation and improvement of cancer treatment methods, the overall survival rate and prognosis of cancer patients have been significantly improved. However, the recurrence of tumors is always inevitable. The cancer therapy has encountered a bottleneck. An emerging concept is that tumor proliferation and growth are also strongly dependent on external signals from their microenvironment (5–7). Therefore, in order to fully understand tumor development and progression, a deeper understanding of the interaction between cancer cells and their microenvironment is needed. The role of statins in tumors is also being studied. However, the role of statins in the tumor microenvironment has rarely been systematically described, we hope that this brief review will shed some light on the role of statins in the tumor microenvironment and suggest further progress.

## Summary of Studies on the Direct Anti-Tumor Mechanism of Statins

### Statins Exert Anti-Tumor Effects by Inhibiting the Metabolites of the Mevalonate Pathway

**Figure 1:**
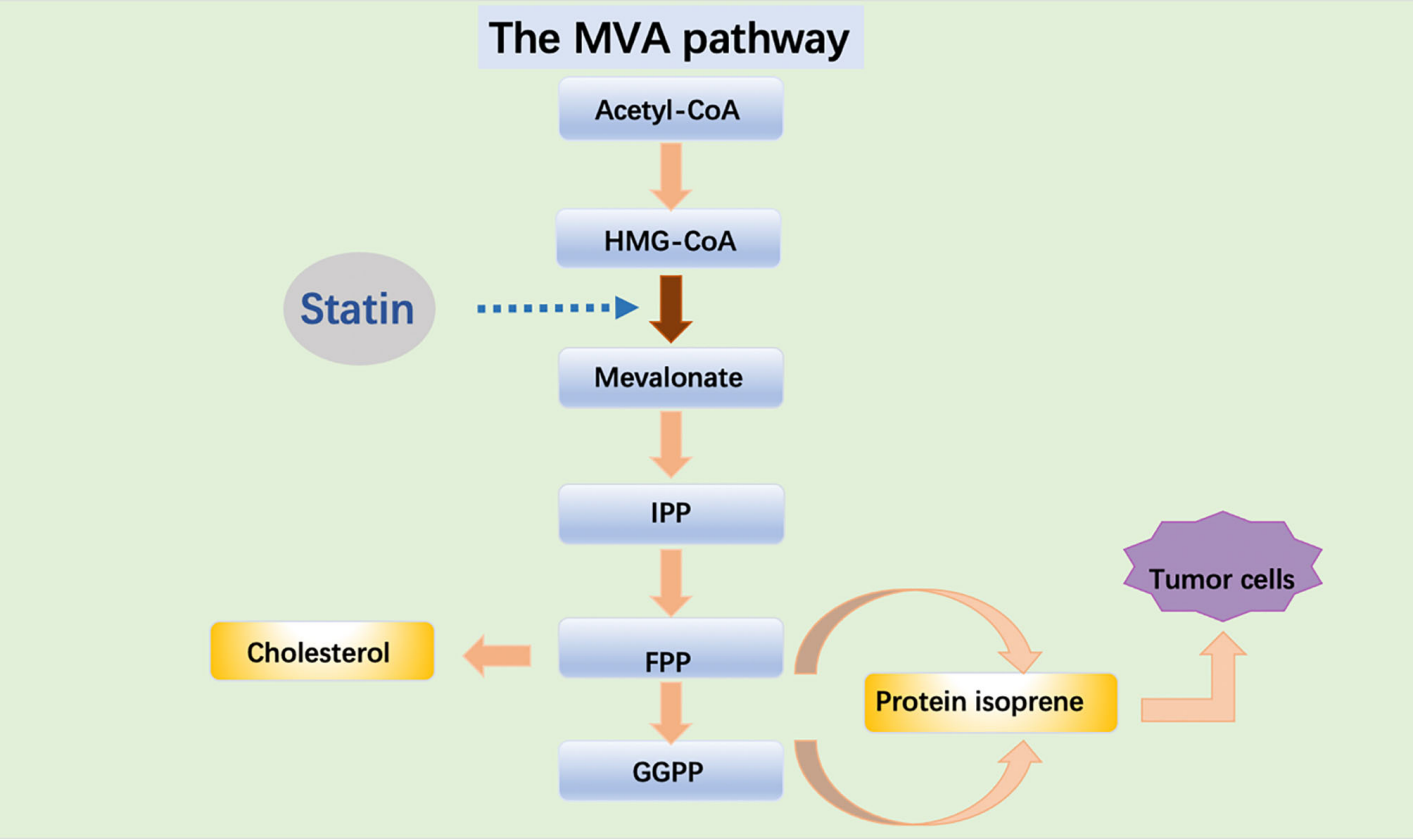
Acetyl-CoA is transformed into HMG-CoA which is used by the enzyme HMGCR to synthesize MVA. MVA is further metabolized to farnesyl pyrophosphate (FPP), a precursor of cholesterol and sterols. FPP is also converted to geranylgeranyl pyrophosphate (GGPP), and these lipids are used for post-translational modification of proteins, including N-glycosylation and protein prenylation.

Statins can downregulate blood lipid levels and play a role in the prevention and treatment of cardiovascular diseases. Statins are being investigated to prevent and treat cancer. Of all the mechanisms studied, the most widely studied is the mevalonate pathway. The mevalonate pathway is an essential metabolic pathway that produces sterols and isoprene, which are essential for tumor growth and progression (8) (**Figure 1**). In tumors, one of the metabolic adaptations to accelerate tumor growth is the upregulation of the valerate pathway, which is associated with the origin, progression and phenotype of malignant tumors (8, 9). Cholesterol plays an important role in cell proliferation and cell cycle and plays a central role in the lipid raft (10). The proliferation of tumor cells is dependent on cholesterol and cancer cells maintain high intracellular cholesterol through different mechanisms (11). Cholesterol directly activates the oncogenic Hedgehog pathway and induces mechanistic target of rapamycin complex 1 (mTORC1) signaling pathway to promote cancer (12). Cancer cells have a higher cholesterol requirement than normal cells and contain more lipid rafts to meet the need for tumor-promoting cell signaling proteins (13). Statins exert anti-proliferative, anti-angiogenic, pro-apoptotic, and anti-metastatic action on cancer cells *via* inhibition of both isoprenoid and cholesterol synthesis in the MVA pathway (14).

### Statins Are Involved in Ferroptosis

Ferroptosis is a novel type of programmed cell death, which is associated with the development of many diseases, including cancer (15). Ferroptosis is characterized by iron overload, lipid reactive oxygen species (ROS) accumulation, and lipid peroxidation (16). Two main pathways of ferroptosis have been elucidated. Among the main pathways, glutathione peroxidase 4 (GPX4)-glutathione (GSH) -cysteine axis, GPX4-related lipid peroxidation, and System Xc-, the upstream node of GPX4-GSH-cysteine axis, are important pathways that trigger ferroptosis (17, 18).

**Figure 2:**
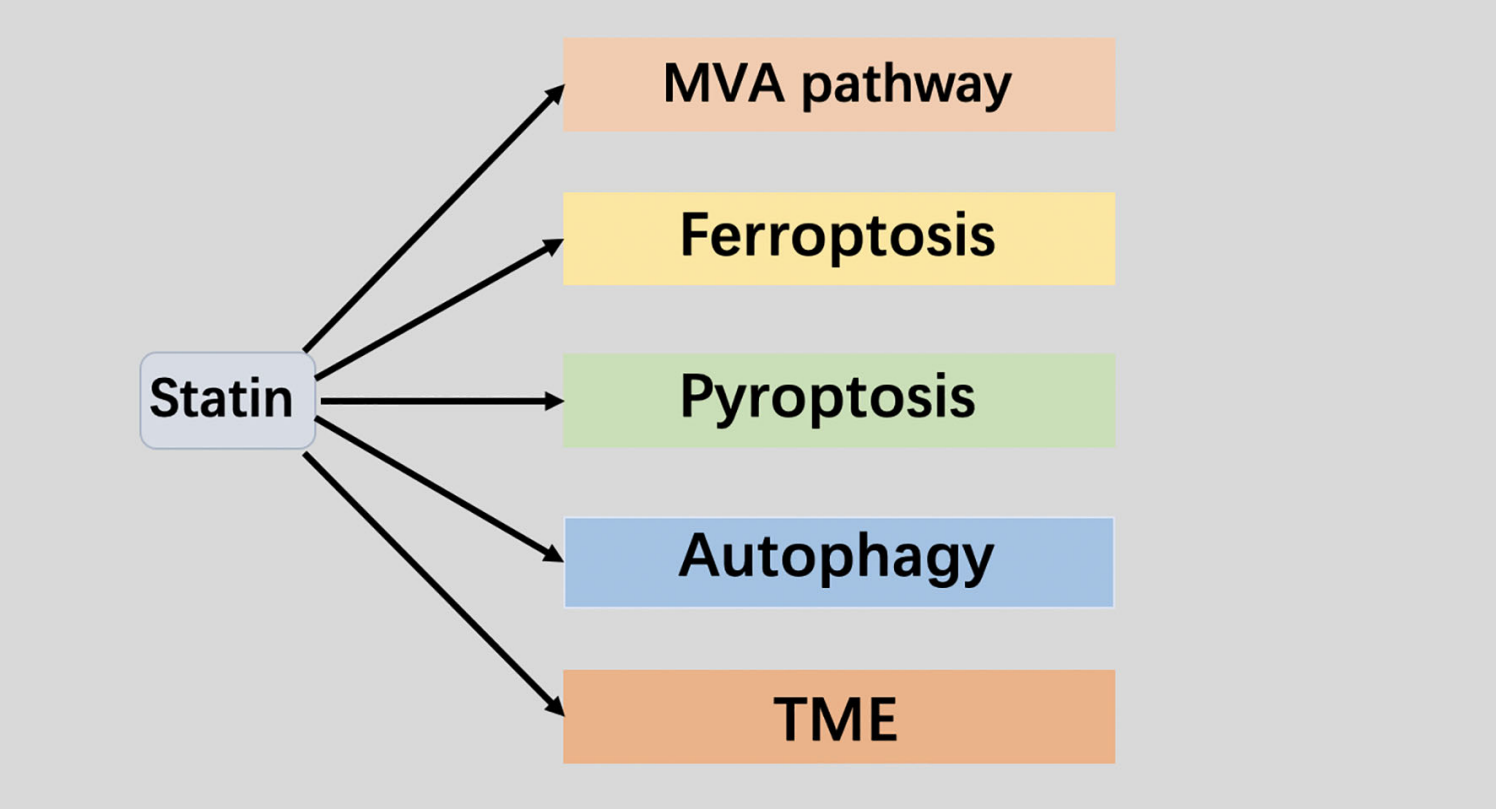
Statin targets in cancer therapy.

Studies have shown that ferroptosis is related to mevalonate pathway metabolism, which directly impinge on the cells’ sensitivity toward lipid peroxidation and ferroptosis (19). GPX4 is a selenoprotein, which containing the REDOX active center of selenocysteine (20). The transport of selenocysteine requires special selenocysteine tRNA (SEC-TRNA) (21). The isopentene group, which is important for the function of sec-tRNA, is derived from the IPP of the mevalonate pathway (22). Therefore, inhibitors of the Mevalonate pathway will block the maturation of selenocysteine tRNA and the synthesis of GPX4 (23). In addition, another product of the Mevalonate pathway is Coenzyme Q10 (CoQ10), a powerful antioxidant in membranes, can represses ferroptosis under the oxidative stress (24). Therefore, statins may be a valuable candidate for inducing ferroptosis in tumor (**Figure 2**).

### Statins Are Involved in Pyroptosis

Pyroptosis is a type of programmed cell death associated with inflammation, and it was first described in 1992 in myeloid cells infected with pathogens or bacteria (25, 26). It is primarily triggered by inflammasome and is performed by the caspases and Gasdermin protein families, including gasderminA (GSDMA), gasderminB (GSDMB), gasderminC (GSDMC), gasderminD (GSDMD), and gasderminE (GSDME). More and more evidences indicate that pyrotopia plays an important role in regulating cancer progression. The pyroptosis can be induced through two main pathways, including the canonical caspase-1 inflammasome pathways and non-canonical caspase-4/5/11 inflammasome pathways (27–29). Both pyrophosis pathways eventually induce cell membrane pore formation, cell membrane rupture, and cell death (30).

Many studies have shown that the inhibition of pyrophosis by statins is associated with the occurrence and progression of diseases, such as non-alcoholic fatty liver disease (NAFLD) and coronary microembolism (CME) (31, 32). However, the potential association of pyrophosis in the antitumor effects of statins remains to be fully elucidated. Simvastatin has been shown to induce pyrophosis in xenograft mouse models and non-small cell lung cancer (NSCLC) cell lines. Fortunately, simvastatin treatment reduced tumor cell viability and migration without causing toxicity to normal lung cells (33). The mechanism of statins and pyrophosis in tumors still needs to be further explored.

### Statins Regulate Autophagy

Autophagy is a conserved catabolic process essential for homeostasis, and it delivers intracellular proteins, lipids and organelles to the lysosomal compartment where they will be degraded (34–36). In cancer, the role of autophagy is one of a double-edged sword. On the one hand, autophagy enables tumor cells to tolerate stress including a hypoxic microenvironment and starvation. On the other hand, autophagy plays an important role in damage mitigation which can limit tumorigenesis (37). Therefore, the regulation of autophagy is undoubtedly a feasible strategy in the treatment of tumors.

Studies have revealed that simvastatin regulates autophagy flux and promotes cancer cell death by stimulating the ERK1/2 (38). Combined treatment of tumor cells with Farnesyltransferase inhibitor and lovastatin resulted in effective autophagy (39). Lovastatin stimulates autophagy through the Rac/phospholipase C/inositol 1,4,5-triphosphate axis, which reduces the viability and migration of malignant pleural mesothelioma cells (40). Therefore, regulating autophagy may be a promising tumor therapy. There is no doubt that statin maybe a valuable candidate.

## Statin and Tumor Microenvironment

### Lipid Metabolism Disorders in Cancer Patients

The World Health Organization (WHO) estimates that more than 1 billion adults are overweight, with more than 300 millions of them considered obese. Clinical evidence shows that obesity is associated with an increased incidence of certain malignant tumors, such as liver, colon, pancreas, breast, etc (41). For cells, lipids not only provide sufficient building blocks for rapid membrane formation, but also generate signaling molecules and substrates for post-translational modification of proteins (42). Lipid metabolism is also essential for cancer progression (43). Disruption of lipid metabolism has been found in many types of tumors, including pancreatic cancer, prostate adenocarcinoma, and lung cancer (43–45). For example, in pancreatic cancer, lipids can adequately stimulate the proliferation of pancreatic cancer cell lines (46). Many enzymes involved in *de novo* fatty acid and cholesterol synthesis were significantly upregulated in pancreatic cancer (47).

### Lipid Metabolic Abnormalities in the Metabolic Microenvironment

The study of lipid metabolism in early tumor focuses on tumor cells. In recent years, more and more studies have found that lipid metabolism plays an important role in tumor microenvironment in the process of tumor cell proliferation (48). Cancer is typically characterized by increased glucose, lipid, glutamine, and amino acid metabolism, lactic acid accumulation, and ROS dependence. Lipids, which include cholesterol, fatty acids and triglycerides, are responsible for the production of cancer cell membranes, post-translational modifications of proteins, and energy for cancer cells (49). A common feature of cancer cells is their ability to rewire metabolism to maintain the production of ATP and macromolecules needed for cell growth, division, and survival (50). The role of fatty acids as essential mediators for cancer progression and metastasis by reshaping the tumor metabolic microenvironment (50). Oncogenic signaling pathways play a key role in shaping the tumor metabolic microenvironment by directly regulating metabolic enzymes involved in lipid metabolism (50). Due to the rapid proliferation of cancer cells, they require high levels of cholesterol, so an increase in cholesterol biosynthesis is necessary for many tumors (51). In short, cancer cells are hungry, and their metabolism changes based on the tumor microenvironment to meet the demands of cells and nutrients that cancer cells need to grow rapidly.

### The Role of Statin in the Microenvironment of Tumor Metabolism

Metabolic reprogramming is thought to be one of the characteristics of tumor cells and is associated with genomic instability, chronic inflammation that causes tumors, and immune system escape (52). Statins affect the metabolism microenvironment. Simvastatin induces metabolic reprogramming of tumor cells, reduces lactic acid production, and promotes tumor sensitivity to monocarboxylate transporter 1 (MCT1) inhibitors, synergistic anti-tumor effects (53). Furthermore, SREBPs are a family of membrane-bound transcription factors in the endoplasmic reticulum, which play a central role in the regulation of lipid metabolism. Recent studies have shown that SREBPs are highly up-regulated in a variety of cancers and promote tumor growth (54). The PI3K/Akt/mTOR pathway is often overactivated in cancer and plays an important role in tumor cell growth and survival (55). PI3K-AKT-mTORC1-SREBP signal enhances AKT signal in human melanoma cells. Inhibition of the SREBP pathway weakens the activation of AkT in lipid rafts and inhibits the growth of human melanoma cells. These results suggest that PI3K-Akt-mTORC1 signaling plays an important role in tumor cell growth and reproduction by regulating SREBP activation and subsequent cholesterol generation (56). Recent studies have found that statins inhibit the PI3K/Akt/mTOR signaling pathway in colorectal cancer (57). Targeting statins may be a new cancer treatment strategy. MVA pathway gene expression is mainly controlled by SREBP transcription factor (8). Inhibition of SREBP transcription factor increases the efficacy of valerate pathway inhibitors as anticancer agents and may counter drug resistance (8).

**Figure 3:**
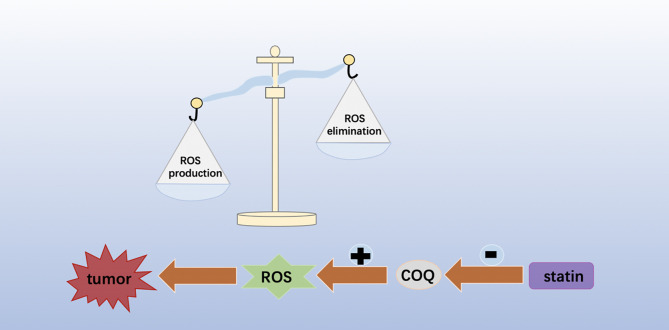
Statins can mediate the loss of COQ and lead to severe oxidative stress, resulting in significant ROS production, which helps to improve the efficacy of chemotherapy and shows anti-tumor activity.

ROS are generally considered by- products of oxygen consumption and cellular metabolism, formed by the partial reduction of molecular oxygen (58, 59). ROS has been shown to be associated with cancer, and increased ROS levels are thought to be carcinogenic, causing damage to DNA, proteins and lipids, and promoting genetic instability and tumorigenesis (**Figure 3**). ROS also plays a role as signaling molecules in cancer, promoting abnormal cell growth, metastasis, resistance to apoptosis, angiogenesis, and also plays a role of differentiation arrest in some types of cancer. However, toxic levels of more ROS in cancer have anti-tumor effects, leading to increased oxidative stress and induction of tumor cell death (60–62). A new study has found that statins can mediate the loss of COQ and lead to severe oxidative stress, resulting in significant ROS production, which helps to improve the efficacy of chemotherapy and shows anti-tumor activity (63). Therefore, therapies designed to eliminate ROS or increase ROS production could be potentially effective cancer therapies.

## Other Tumor Microenvironments and Statins

### Anoxic Microenvironment

HIF-1 is a key factor regulating cell adaptation to hypoxia (64, 65). In pancreatic cancer cells, it is characterized by a highly hypoxic microenvironment (66). A large number of studies have shown that HIF-1α plays a central role in the carcinogenesis and progression of pancreatic cancer, and can promote the proliferation, invasion and metastasis of tumor cells. It also mediates tumor growth, angiogenesis and immune escape (67). In addition, the production of HIF-1 in these malignant cells is further amplified by the hypoxic tumor microenvironment, suggesting that, under hypoxia, HIF-1 may activate different signaling pathways to promote cell survival and proliferation (68). Loss of HIF-1alpha reduces hypoxia-induced vascular endothelial growth factor expression and impairs vascular function, leading to a hypoxic microenvironment within tumor masses (69). Some observations have shown that the MVA pathway can be directly or indirectly regulated under hypoxia, in part because HMGCR expression is regulated through the transcriptional activity of HIF-1α (70, 71). Similarly, fluvastatin stimulates HIF-1α ubiquitin/proteasome degradation (72). Furthermore, in breast cancer, simvastatin activation of AMPK suppresses breast tumor angiogenesis by blocking HIF-1α-induced pro-angiogenic factors (73). In acute myeloid leukemia, potent statins selectively enhance the effects of the cellular poison cytarabine. In a hypoxic environment, combination therapy based on more potent statins enhances targeting of residual acute myeloid leukemia (AML) cells (74). In addition, the anticancer strategy of statins combined with cytotoxic drugs in anoxic environments is valuable in melanoma, where these drugs neutralize the structural expression of HIF-1α in the tumor (75). In conclusion, we believe that statins are likely to be effective anti-tumor drugs in the hypoxic microenvironment of tumors. Its antitumor mechanism in anoxic microenvironment still needs to be further explored.

### Immune Microenvironment

Tumor immune microenvironment is a complex system, one of its core features is immune function (76). *In vivo*, the recruitment of immune cells in the adjacent environment of the tumor is active and forms a different immune environment, thus having a profound impact on clinical outcomes. For example, T-cell activation includes positive and negative checkpoint signals to fine-regulate responses and prevent excessive pathological changes (77, 78). Tumor immune microenvironment contains a variety of immune cells, such as T lymphocytes, B lymphocytes, natural killer cells, mast cells, myeloid suppressor cells, etc. (79). Tumor infiltrating CD8+ T cells play a key role in anti-tumor activity. Tumor-infiltrating CD8+ T cells are associated with progressive loss of effector function due to prolonged antigen exposure and inhibitory tumor microenvironment (80). The dysfunctional state of CD8+ T cells is known as exhaustion, and exhausted CD8+ T cells have high expression of inhibitory receptors such as programmed death -1(PD-1), LAG-3, TIM-3, 2B4, and CTLA-4 (80). However, the mechanism by which infiltrating T cells lose their antitumor activity remains unclear. One result showed that the tumor microenvironment was rich in cholesterol and that cholesterol induced immune checkpoint expression and failure of CD8 + T cells, and statin lowers PD-1 expression in CD8^+^ T cells and effectively restores antitumor activity (81). Statins have also been reported to target the immune microenvironment through cytokines or chemokines. Statins disrupt communication between cancer cells and mesenchymal stromal cells (MSC) by inhibiting CCL3 secreted by cancer cells and IL-6 and CCL2 produced by MSC. This phenomenon inhibits lung cancer cell survival, suggesting a re-use of statins in targeting the immune microenvironment (82). In colon cancer, the combination of statins and difluoromethylornithine (DFMO) increases the activity of functional NK cells, showing a significant inhibitory effect on colon adenocarcinoma (83). In melanoma, statin therapy induces MHC class I Chain-related protein A (MICA) overexpression and increases the sensitivity of NK cells to tumor cell killing (84). All the above findings support the anti-tumor potential of statins. Targeted immune microenvironment immunity provides selectivity for the treatment of tumors in the future, and statins may become a new targeted drug in tumor treatment in the future.

### Acid Microenvironment

Acid microenvironment plays an important role in tumor proliferation, invasion, migration, immune escape and other processes, and affects the effective treatment of tumors (85, 86). As mentioned above, the tumor microenvironment is an anoxic state. Under hypoxia, tumor cells can enhance the expression of glycolysis gene by upregulating HIF-1α and promote the formation of acidic microenvironment (87). It has been found in a variety of tumors that fatty acid synthesis increases along with the accumulation of H+, which contributes to the generation of acidic tumor microenvironment (88). Statins significantly reduced plasma free fatty acid concentrations (89). In cancer cells, activation of epithelial-mesenchymal transition (EMT) leads cells to acquire migration, invasion, and drug resistance (90). Tumor microenvironment is a factor mediating EMT-driven drug resistance (91). Several reports suggest that environmental acidosis stimulates EMT, during which epithelial cells lose their polarity and cell–cell adhesions and become mesenchymal like with enhanced migratory and invasive properties (86). In pancreatic cancer cells, acid stimulation leads to activation of EMT (92). After 24 hours of extracellular acidosis, the expression of EMT markers in melanoma cells was increased and the invasiveness *in vitro* was enhanced (93). Transforming growth factor beta (TGFβ) plays an extremely important role in inducing EMT formation. TGFβ induces EMT through SMAD-mediated and non-SMAD signal (94). Atorvastatin can partially inhibit TGF-β1-induced EMT in non-small cell lung cancer cells by decreasing the up-regulation of Sphk1 (95).

### Mechanical Microenvironment

**Figure 4:**
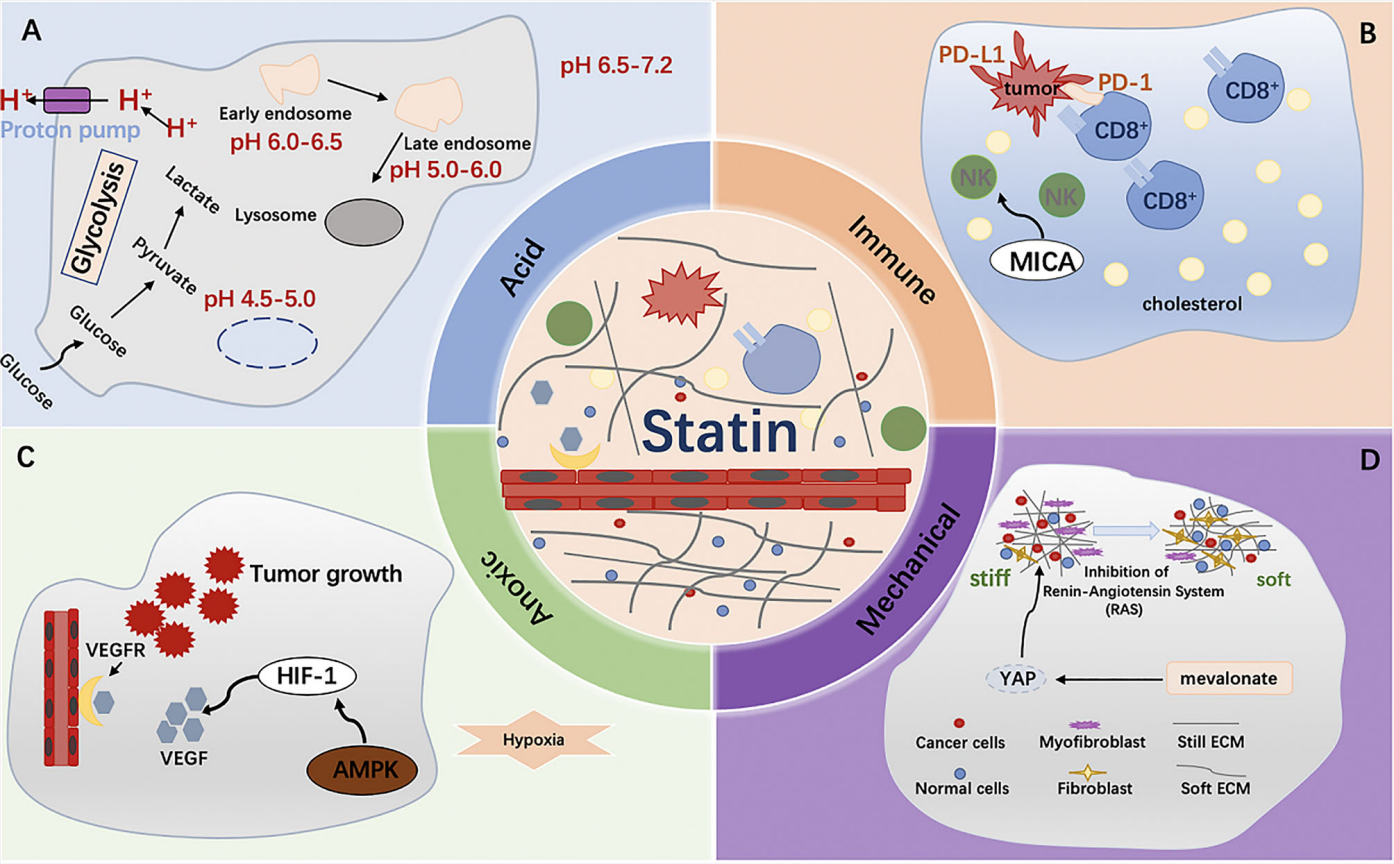
**(A)** Fatty acid synthesis increases along with the accumulation of H+, which contributes to the generation of acidic tumor microenvironment. Statins significantly reduced plasma free fatty acid concentrations; **(B)** Tumor microenvironment was rich in cholesterol and that cholesterol induced immune checkpoint expression and failure of CD8 + T cells, and statin lowers PD-1 expression in CD8+ T cells and effectively restores antitumor activity; **(C)** HIF-1α plays a central role in the carcinogenesis and progression of cancer, and can promote the proliferation, invasion and metastasis of tumor cells. In breast cancer, simvastatin activation of AMPK suppresses breast tumor angiogenesis by blocking HIF-1α-induced pro-angiogenic factors; **(D)** Activation of YAP can control the expression of various cytoskeleton regulatory factors, harden the surrounding matrix of fibroblasts, and promote the invasion of cancer cells.

In recent years, mechanical microenvironment has been found to have an important relationship with the occurrence and development of tumors, which is another special microenvironment that has been studied in recent years (96) (**Figure 4**). There are indications that the tumor exhibits a higher cell density, making it stiffer and significantly different from normal tissue (97). Tumor mechanical microenvironment plays a role in promoting malignant transformation of cells and also affects the sensitivity of tumor cells to therapeutic drugs (97). In 1971, Judah Folkman first emphasized that angiogenesis is essential for the growth and proliferation of solid tumors, and therefore anti-tumor angiogenesis is a feasible approach for the treatment of solid tumors (98, 99). Currently, many anti-angiogenic drugs, such as Avastin and Ramucirumab, have achieved remarkable results in the treatment of cancer (100). Highly activated metastasis-related fibroblasts increase tissue stiffness, thereby increasing resistance to angiogenesis and antiangiogenic therapy. In a study of primary colorectal cancer with liver metastasis, inhibition of fibroblast contraction and extracellular matrix deposition decreased liver metastatic tumor hardening and enhanced the antiangiogenic effects of bevacizumab (101). And patients receiving bevacizumab combined with renin-angiotensin inhibitors had an increased overall survival (101). Simvastatin, an effective statin, attenuated Ang II-induced cardiac fibrosis (102). Therefore, we hypothesized that targeting Ang II with statins to induce cell fibrosis might be an effective strategy. The specific mechanism is worth exploring. Among the stromal cells that make up the tumor microenvironment, cancer-associated fibroblasts (CAFs) are the most abundant and are closely associated with cancer progression (103). The presence of fibroblasts in tumor stroma, also known as cancer-associated fibroblasts, plays an important role in the regulation of tumor tissue hardening. Activation of YAP can control the expression of various cytoskeleton regulatory factors, harden the surrounding matrix of fibroblasts, and promote the invasion of cancer cells (97). Transcriptional regulators YAP and TAZ have attracted much attention due to their significant biological properties in development, tissue homeostasis, and cancer (104). In addition, YAP/TAZ activity was controlled by the SREBP/mevalonate pathway. Statins can inhibit the rate-limiting enzyme (HMG-CoA reductase) of this pathway, and reduce the transcriptional activity of YAP/TAZ and thus have an anti-tumor effect (105).

## Targets of Statins in the Tumor Microenvironment

HHIF-1 activates the transcription of genes that are involved in key aspects of cancer biology, including angiogenesis, cell survival, glucose metabolism and invasion. The hypoxic microenvironment in tumors can lead to the overexpression of HIF-1α. In preclinical studies, inhibition of HIF-1 activity has a significant effect on tumor growth (106). In addition, in hypoxic microenvironment, hypoxic-inducible factor activation leads to tumor metabolic reprogramming, may activate different signaling pathways to promote tumor proliferation, and contribute to the immunosuppression of tumor microenvironment (67, 68).

The tumor microenvironment is rich in cholesterol, which induces CD8+ T cell immune checkpoint expression and failure, and statins reduce CD8+ T cell PD-1 expression, effectively restoring anti-tumor activity (81). In addition, Statins restrict tumor cell growth by inhibiting CCL3 secreted by cancer cells and IL-6 and CCL2 produced by MSC, thereby disrupting the communication between cancer cells and MSC (82).

EMT is the process by which epithelial cells acquire mesenchymal characteristics. In cancer, EMT is associated with tumor initiation, invasion, metastasis, and therapeutic resistance (107). EMT is stimulated by environmental acids, during which epithelial cells lose polarity and intercellular adhesion and become mesenchymal, with enhanced migration and invasion. TGFβ plays an important role in inducing the formation of EMT. Statins partially inhibit TGF-β1-induced EMT in non-small cell lung cancer cells by decreasing the upregulation of sphingosine kinase 1 (SpHK1) (95).

YAP/TAZ is a transcriptional coactivator that travels between cytoplasm and nucleus and is an effector of hippopotamus signaling cascade, stimulating the genesis, development and metastasis of tumors (104, 108). Activation of YAP can control the expression of various cytoskeleton regulators, harden the surrounding stroma of fibroblasts, and promote the invasion of cancer cells. YAP/TAZ activity is controlled by the SREBP/mevalonate pathway. Statins can inhibit the rate-limiting enzyme (HMG-CoA reductase) of this pathway and reduce the transcriptional activity of YAP/TAZ, thus having an anti-tumor effect. Thus, YAP/TAZ is an attractive therapeutic target in cancer.

## Statins Reverse Drug Resistance by Regulating Tumor Microenvironment

Resistance to anticancer drugs in human tumors is often attributed to gene mutations, gene amplification, or epigenetic changes that affect uptake, metabolism, or export of single-cell drugs, and a large body of evidence suggests that mechanisms involved in the tumor microenvironment also mediate resistance to chemotherapy in solid tumors (109). TME reduces drug penetration, confers survival cell proliferation and antiapoptotic advantages, and promotes resistance without causing genetic mutations and epigenetic changes (110). The vascular system affects a tumor’s sensitivity to drugs because cancer drugs enter the tumor through the bloodstream (111). Solid tumor is a tissue with abundant blood supply, but the blood vessels in tumor tissue are different from those in normal tissue in function and structure (112, 113). For many solid tumors, antitumor angiogenic therapy offers greater clinical benefit when used in combination with chemotherapy. This is due in part to normalization of the tumor vasculature, resulting in improved delivery and efficacy of cytotoxic drugs (113, 114). Statins have been shown to reduce the production of vascular endothelial growth factor and inhibit capillary formation (115–117). Finally, there is growing evidence that the effect of statins on angiogenesis is concentration-dependent. Weis et al. showed that low concentrations (0.5 mg/kg per day) of cerivastatin and atorvastatin enhanced endothelial cell proliferation, but high concentrations (2.5 mg/kg per day) significantly inhibited angiogenesis (118). Hypoxia in tumors will lead to the activation of genes related to angiogenesis and cell survival, and the expression of these genes may promote the proliferation of tumor cells and produce drug resistance (119, 120). The pH in the tumor microenvironment affects the cytotoxicity of anticancer drugs (109). Drugs that improve drug delivery or activity by targeting the tumor’s microenvironment are an important future direction for cancer therapy. Moreover, the optimal dose concentration of statins still needs to be further explored.

## The Role of Statins Combined With Radiotherapy, Chemotherapy and Immunotherapy in Anti-Tumor

Preclinical evidence shows that statins have a significant effect on radiotherapy, chemotherapy, and immunotherapy. Statins have been found to increase the sensitivity of patients to radiotherapy while treating tumors. *In vitro* efficacy of lovastatin combined with radiotherapy for B-cell rat lymphoma (L-TACB) shows a new role for statins as radiosensitizers. This effect may be the result of enhanced apoptosis (121). Simvastatin treatment can inhibit the viability of colorectal cancer (CRC) cells and enhance the sensitivity to external radiation. These effects may be related to depletion of GGPP and decreased levels of ERK1/2 phosphorylation, suggesting an important role of statins in enhancing radiosensitivity through the EGFR-Ras-ERK1/2 pathway (122).

Lovastatin can enhance the effectiveness of chemotherapy drugs in the treatment of malignant melanoma (123). The data suggest that lovastatin may be combined with 5-Fu or cisplatin to enhance antitumor activity in colon cancer (124). Statins can increase the sensitivity of gemcitabine - erlotinib chemotherapy to cancer treatment (125). Tumor immune escape refers to the phenomenon that tumor cells grow and metastasize through various mechanisms to escape the recognition and attack of the immune system, which is an important strategy for the survival and development of tumors (126).

The programmed death ligand-1 (PD-L1)/PD-1 signaling pathway inhibits the activation of T lymphocytes and enhances the immune tolerance of tumor cells, thus realizing tumor genesis and immune escape. Thus, targeting the PD-L1/PD-1 pathway is an attractive cancer treatment strategy (126). However, the efficacy of PD-1/PD-L1 antagonists in solid tumors, including lung cancer and colorectal cancer, is currently unsatisfactory, possibly due to the complex tumor microenvironment (126, 127). According to the latest research, in malignant pleural mesothelioma and advanced non-small cell lung cancer, combination of statins with PD-1 inhibitors results in better clinical outcomes (128). The possible mechanism is that statins interfere with the prenylation of small GTPase proteins, thereby preventing intracellular vesicle transport, resulting in prolonged antigen retention on the cell membrane and enhanced antigen presentation to T cells (129).

## Discussion

In this review, we summarize the mechanism of antitumor action of statins. Clinical trials have shown that some patients respond to statins, but not all. In conclusion, we reanalyzed the antitumor effects of statins in tumors from the perspective of tumor microenvironment. Statins are a potential antitumor drug. The specific mechanisms and potential targets of anti-tumor in the tumor microenvironment still need long-term research to promote more precise and individualized treatment. We need to find more valuable biomarkers to identify responders and develop combination therapies to further improve their effectiveness.

## Author Contributions

P-FZ and M-XW contributed equally to this work. Z-LC and LY revised the manuscript. All authors wrote and revised the manuscript and issued the final approval for the version to be submitted.

## Conflict of Interest

The authors declare that the research was conducted in the absence of any commercial or financial relationships that could be construed as a potential conflict of interest.

## Publisher’s Note

All claims expressed in this article are solely those of the authors and do not necessarily represent those of their affiliated organizations, or those of the publisher, the editors and the reviewers. Any product that may be evaluated in this article, or claim that may be made by its manufacturer, is not guaranteed or endorsed by the publisher.
